# Evaluation of Photoelectrocatalysis with Electrode Based on Ti/RuO_2_-TiO_2_ Modified with Tin and Tantalum Oxides for the Degradation of Indigo Blue Dye

**DOI:** 10.3390/nano12234301

**Published:** 2022-12-04

**Authors:** Alveriana Tagarro Tomaz, Carla Regina Costa, Maria de Lourdes S. Vasconcellos, Rolando Pedicini, Josimar Ribeiro

**Affiliations:** 1 Laboratorio de Pesquisa e Desenvolvimento em Eletroquímica (LPDE), Department of Chemistry, Center of Exact Sciences, Federal University of Espírito Santo, Campus Goiabeiras, Av. Fernando Ferrari, Vitória 29075-910, Brazil; 2 Departamento de Química, Universidade Federal do Triângulo Mineiro, Uberaba 38025-180, Brazil; 3 Instituto di Tecnologia Avanzate per l’Energia “Nicola Giordano” (ITAE), Via S. Lucia Sopra Contesse, 5-98126 Messina, Italy

**Keywords:** photoelectrocatalysis, Indigo Blue, dimensionally stable anode

## Abstract

Indigo Blue (IB) is a dye widely used by the textile sector for dyeing cellulose cotton fibers and jeans, being considered a recalcitrant substance, and therefore resistant to traditional treatments. Several methodologies are reported in the literature for the removal or degradation of dyes from the aqueous medium, among which photoelectrocatalysis stands out, which presents promising results in the degradation of dyes when a dimensionally stable anode (DSA) is used as a photoanode. In the present work, we sought to investigate the efficiency of a Ti/RuO_2_-TiO_2_ DSA modified with tin and tantalum for the degradation of Indigo Blue dye by photoelectrocatalysis. For this, electrodes were prepared by the thermal decomposition method and then a physical–chemical and electrochemical analysis of the material was carried out. The composition Ti/RuO_2_-TiO_2_-SnO_2_Ta_2_O_5_ (30:40:10:20) was compared to Ti/RuO_2_-TiO_2_ (30:70) in the photocatalysis, electrocatalysis, and photoelectrocatalysis tests. The photocatalysis was able to degrade only 63% of the IB at a concentration of 100 mg L^−1^ in 3 h, whereas the electrocatalysis and photoelectrocatalysis were able to degrade 100% of the IB at the same initial concentration in 65 and 60 min, respectively.

## 1. Introduction

Dyes are organic substances used in several industrial sectors, such as cosmetics [1], paints [2], leather dyeing [3], and especially in the textile sector [4]. The textile industry is responsible for a large amount of highly polluting effluents, with a high organic load. From an environmental point of view, the dyeing stage is the most worrying due to the large number of substances that do not bind to the fibers and generate residues that are released into aquatic environments [5]. Knowing that dyes are potentially toxic pollutants and resistant to conventional treatment systems, it is necessary to study alternative methods [6]. Numerous alternatives to bleach textile effluents have been proposed [7], including studies on Advanced Oxidative Processes (AOPs).

AOPs are methods capable of processing high organic loads and causing less secondary pollution compared to traditional treatment methods [8]. These methods use the in-situ formation of highly reactive oxidizing species that are capable of promoting the mineralization of various organic pollutants, originating substances with low toxic potential [9,10]. The generation of these reactive species can be obtained through several processes, such as electrocatalysis, photocatalysis, and photoelectrocatalysis.

On electrocatalysis, the effluent is subjected to a defined current applied between two electrodes, the anode, and the cathode, in the presence of a supporting electrolyte. In this process, pollutants can be degraded by direct oxidation or indirect oxidation. In direct anodic oxidation, pollutants are first adsorbed onto the anode surface and then degraded by electron transfer. In indirect oxidation, oxidants are generated electrochemically by an anodic or cathodic process, and these are responsible for degrading the pollutant within the solution [10].

Photocatalysis occurs through the photoexcitation of a semiconductor as a result of the absorption of incident electromagnetic radiation. If the energy of the incident photon is greater than the bandgap of the semiconductor, the valence band electrons can be excited to the conduction band, producing electron/hole pairs (e^−^/h^+^). The formed gaps guarantee the electrochemical system high oxidation capacity and can migrate to the surface to react directly with organic compounds or react with H_2_O/OH^−^, forming hydroxyl radicals (HO^•^). In addition, electrons can react with dissolved oxygen and form reactive oxygen species. These radicals are capable of degrading a wide range of recalcitrant organic compounds. However, in the absence of an electron acceptor, photogenerated e^−^/h^+^ pairs can also recombine and release energy in the form of heat, reducing the efficiency of the process [11,12].

Finally, photoelectrocatalysis is a process that employs photocatalysis combined with electrocatalysis. That is, the semiconductor is radiated with an energy greater than its bandgap, and a potential or current is also applied, through an external circuit, allowing the photogeneration of e^-^ which will be continuously driven to the counter electrode to prevent recombination with the h^+^ photogenerated, a typical photocatalysis problem [13,14].

Several materials can be used as photoanodes for the photoelectrocatalysis process. Among them, TiO_2_ stands out, which has low water solubility, chemical and photochemical corrosion resistance, low cost, and availability [15]. However, it has several limitations: the photocatalytic activity of this semiconductor which is limited to UV irradiation, and the rate of recombination of electron-hole pairs in this semiconductor is high, which reduces the efficiency of the reaction [15].

Among the materials commonly applied as photoanodes, dimensionally stable anodes (DSAs) are considered a photoanode option with a high efficiency for photoelectrocatalysis reactions. This material can be defined as an anode that uses a titanium substrate and is commonly coated with oxides of iridium, ruthenium, cobalt, and lead, and these oxides will act as catalysts to intensify the production of oxidizing species. Zirconium, tantalum, and tin oxides are used as stabilizers and modulators [16,17,18].

DSAs exhibit a high electroactive area due to their morphology, and oxidation occurs either by direct exchange of electrons between the contaminant and the electrode surface or by indirect in situ electrogeneration of catalytic species with high oxidizing power [19]. Thus, the material has excellent catalytic activity, corrosion-resistant nature, stability, and high efficiency in the removal of organics in wastewater [19].

In the literature, some works report the application of DSAs for dye oxidation. Chen et al. used DSA of composition Ti/SnO_2_–RuO_2_ in the degradation of the dye Alizarin Cyanin green, where the process demonstrated good performance and decolorization efficiency of 80.4% [20]. SnO_2_ is a good material to be applied in DSAs due to its high stability corrosion [21], and in this way, it has also been used by Bravo-Yumi et al. (2020), combined with oxide of iridium and antimony, for the degradation of the dyes: Green A, Brown DR, and Violet RL, where the discoloration was greater than 86% in all cases after 6 min of processing [22].

Tantalum oxide was also studied in the degradation of dyes, Niu et al. conducted research using Ta_2_O_5_ photocatalysts. The material was recycled from recycled capacitors and was decorated with polyaniline. It was used in the degradation of Rhodamine B and exhibited excellent photostability and reusability [23]. In turn, Vercesi et al. (1990) studied tantalum oxide, and it was found that the material has excellent properties as a semiconductor and stabilizer and can be used in DSA-type electrodes [24]. As Brazil is the main producer of this metal, it is interesting to transform tantalum into a technological product [25], such as electrodes.

Among the dyes most used by the industry, we have Indigo Blue (IB), widely used for dyeing cellulose cotton and jeans fibers, being considered a recalcitrant substance, which causes environmental concern [26].

IB discoloration was studied by Balan and Monteiro (2001) using ligninolytic basidiomycete fungi from Brazil. Although 100% of the dye was degraded, the process took 4 days [26]. It was also studied using photocatalysis with Fenton’s reagent. In 45 min, the concentration of total organic carbon remained constant, and the degradation was 61%. The initial dye concentration was 25 ppm, where the procedure was performed with a UV lamp with a power of 90 W [27]. The electrochemical decolorization process has also been studied for IB by Sanromán et al. (2004), the degradation of 90% of the dye was obtained in 60 min using a platinum electrode (Vigo, GPo, Spain) at a constant voltage of 5 V at pH 4.5 and an initial concentration of 100 μM at 25 °C [28].

Due to the wide use of IB dye and the reduced amount of work studying its discoloration by electrochemical means, this article aims to study its degradation by means of photoelectrocatalysis using an electrode based on Ti/TiO_2_-RuO_2_ modified with tin and tantalum.

## 2. Experimental

### 2.1. Preparation of Electrodes

The electrodes of composition Ti/RuO_2_-TiO_2_-SnO_2_-Ta_2_O_5_ with the nominal atomic percentage of 30:40:10:20, Ti/RuO_2_-TiO_2_-SnO_2_-Ta_2_O_5_; 30:50:10:10 and RuO_2_TiO_2_ 30:70 were prepared by the thermal decomposition method. The three precursor solutions were prepared with 0.1 mol L^−1^ of ruthenium chloride(III) (Dinâmica^®^, Serra, ES, Brazil), titanium isopropoxide(IV) (Dinâmica^®^, Serra, ES, Brazil), tin chloride (Dinâmica^®^, Serra, ES, Brazil), and tantalum ethoxide(V) (Dinâmica^®^, Serra, ES, Brazil) in ethanol (Dinâmica^®^, Serra, ES, Brazil). The temperature of these solutions was controlled between 60 and 65 °C and a mixture of citric acid (Dinâmica^®^, Serra, ES, Brazil) and ethylene glycol (Dinâmica^®^, Serra, ES, Brazil) was added and heated to 80–95 °C to promote the esterification reaction. The mixture was kept in this temperature range for 1 to 2 h until the volume of the solution was reduced to half.

The 21 cm^2^ Ti supports, previously blasted with a glass microsphere using the GJS blaster (PR Jateamento^®^, Boa Esperança do Sul, SP, Brazil) were washed with running water and kept in hot ultrapure water for 30 min. Then, they were immersed in a beaker containing 99.5% pure isopropyl alcohol (NEON^®^, Suzano, SP, Brazil), and placed in the USC-1400 ultrasound (UNIQUE^®^, Indaiatuba, Brazil) with a power of 135 Watts RMS for 30 min to degrease their surface. Subsequently, the Ti supports were placed in a 20% HCl solution (NEON^®^, Suzano, SP, Brazil) at boiling point for 30 min, and subjected to a chemical attack by 10% oxalic acid (Dinâmica^®^, Serra, ES, Brazil) for 20 min. The electrodes were dried in a Toyo TA-1060 heat gun (Toyo^®^, Curitiba, PR, Brazil), weighed, and brushed with polymeric resins. After depositing the material, the resin was dried, taken to an oven at 130 °C for 10 min, and then to a muffle furnace at 450 °C for 5 min until reaching a mass of 2 mg. Upon reaching the desired mass, the electrode was calcined for 1 h at 450 °C.

### 2.2. Characterization of Electrodes

#### 2.2.1. Physicochemical Characterization

In order to analyze the formation of oxides formed during the electrode calcination process and to determine the crystalline phases present, a study of the film by X-ray diffraction (XRD) was carried out. Measurements were performed under a grazing angle, measuring interval 2θ = 10° to 90° in the interval 0.01° min^−1^, using a Shimadzu diffractometer (Belo Horizonte, MG, Brazil), model XRD-6000 SSC with a Kα-Cu radiation source (λ = 1.5406 Å) of the Core Competencies in Petroleum Chemistry (Labpetro).

Other techniques used were scanning electron microscopy (SEM) (São Paulo, SP, Brazil) and Energy scattering X-ray spectroscopy (EDX) (São Paulo, SP, Brazil)to obtain information about the structural properties of the material, as well as its morphology, both from JEOL equipment, model JSM6610LV, with a resolution of 3.0 nm (30 kV), 8 nm (3 kV), 15 nm (1 kV) from the Carlos Alberto Redins Cellular Ultrastructure Laboratory (LUCCAR).

#### 2.2.2. Electrochemical Characterization 

The electrodes were investigated via cyclic voltammetry using the VersaSTAT 4 potentiostat/galvanostat from Princeton Applied Research. The technique allows knowing the redox processes that occur on the surface of the electrodes, making it possible to obtain information on the reversibility of the reaction and on the occurrence or not of reactions parallel to the electron transfer processes, as well as on the charge involved in the process and the reproducibility of the surface [29].

The electrochemical cell used in the experiments consisted of an Ag/AgCl electrode as a reference (ANALION, R682A), the DSA working electrode with composition Ti/RuO_2_-TiO_2_-SnO_2_-Ta_2_O_5_ (30:40:10:20); (30:50:10:10) and RuO_2_TiO_2_ (30:70), and a counter carbon electrode (4 cm^2^). Before each measurement, the system was purged with nitrogen gas (White Martins, Serra, ES, Brazil) for 15 min in order to remove dissolved oxygen in the solution used.

Electrode hydration was performed with fifty successive sweeps in the region of 0.2–1.0 V vs. Ag/AgCl in H_2_SO_4_ (Sigma-Aldrich^®^, Cotia, SP, Brazil) 0.5 mol L^−1^ at 0.05 V s^−1^. Other analyses were performed using two sequential voltammetric cycles at 0.01 V s^−1^ and a potential window of 0.00 to 1.20 V vs. RHE (reference hydrogen electrode). All reagents used were of analytical grade, and the ultrapure water used was obtained by the Sartorius purification Arium^TM^, model MA-UVT (São Bernado do Campo, SP, Brazil) with a resistivity of 18.0 MΩ cm at 22 °C.

In addition, the Ti/RuO_2_-SnO_2_-TiO_2_-Ta_2_O_5_ system was evaluated according to its composition by the stability test at a corresponding density of 750 mA cm^−2^ in a 0.5 mol L^−1^ H_2_SO_4_ solution. The electrode potential was recorded as a function of time and its useful lifetime, considering the time required for the potential to reach 6 V vs. RHE.

### 2.3. IB Dye Degradation Assays

#### 2.3.1. Electrocatalysis

For the electrocatalysis tests, the IB dye concentration was at 100 mg L^−1^, and an AFR source, model FA3010-M was used, providing a current of 0.02 A at 25 °C until the complete degradation of the dye. In the electrochemical cell, DSAs of composition Ti/RuO_2_-TiO_2_-SnO_2_-Ta_2_O_5_ were used, which were compared to the standard electrode of Ti/RuO_2_-TiO_2_, in both cases a graphite electrode with an area of 3.15 cm^2^ was used as the counter electrode and 150 mL of dye solution were used. The aliquots of the solution were taken every 10 min and the samples were analyzed with the aid of a UV-Vis spectrophotometer (Hach DR5000, Jundiaí, SP, Brazil). Color removal percentages were calculated according to Equation (1).

(1) $$\%\;Color\;Removal=\frac{C_{0}-C_{t}}{C_{0}}\times 100$$

Wherein *C*_0,_ and *C_t_* correspond to the dye concentration at time zero and time *t*, respectively.

#### 2.3.2. Photocatalysis

For the photocatalysis tests, an Ultraviolet (UV) light booth, 365 nm wavelength from T&M instruments model CL6i-45S (Brooklin, SP, Brazil) was used, and no current was applied to the process. For these tests, electrodes of composition Ti/RuO_2_-TiO_2_-SnO_2_-Ta_2_O_5_ and Ti/RuO_2_-TiO_2_ were used, a counter carbon electrode, 150 mL of dye solution 100 mg L^−1,^ and the aliquots of the solution were also taken every 10 min and analyzed with the aid of a UV-Vis spectrophotometer (Hach DR5000). Results were expressed as a percentage of color removal, calculated using Equation (1).

#### 2.3.3. Photoelectrocatalysis

For the photoelectrocatalysis test, the radiation emitted by the UV light cabin was associated with a current of 20 mA applied by a source, a volume of 150 mL of the dye IB 100 mg L^−1^ was used with the electrodes of composition Ti/RuO_2_-TiO_2_-SnO_2_-Ta_2_O_5_ and Ti/RuO_2_-TiO_2_. The samples were taken every 10 min for analysis in the spectrophotometer. Equation (1) was also used to analyze the results.

## 3. Results and Discussion

### 3.1. Physicochemical Characterization

#### 3.1.1. X-ray Diffraction

**Figure 1:**
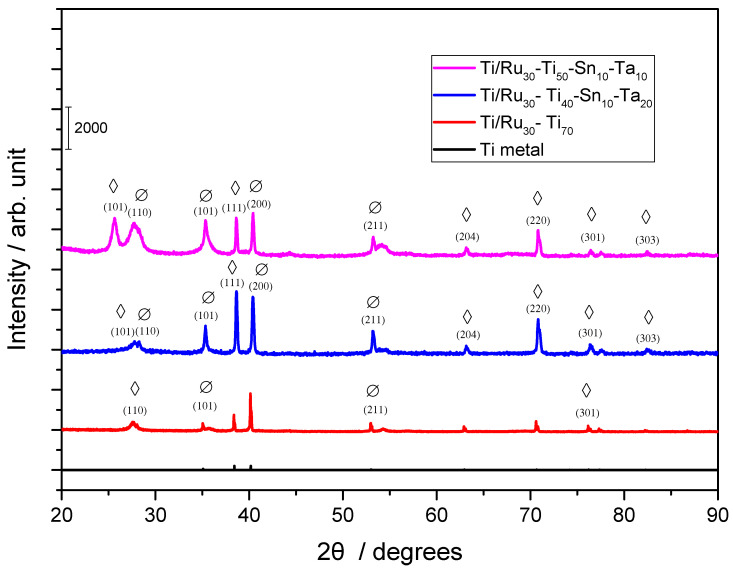
X-ray pattern for the electrodes: Ti/TiO_2_RuO_2_ (70:30), Ti/RuO_2_-TiO_2_-SnO_2_-Ta_2_O_5_ (30:40:10:20) and (30:50:10:10) (Ø) RuO_2_ rutile (PDF 40-1290); (◊) TiO_2_ rutile (PDF 21-1272).

The surface morphology of the quaternary electrodes, of composition Ti/RuO_2_-TiO_2_-SnO_2_-Ta_2_O_5_, was analyzed by X-ray Diffraction (XRD) in order to verify the oxides present, and the result is shown in Figure 1.

Comparing the positions of the peaks obtained with the values established for metallic titanium and for the Ru, Ti, Sn, and Ta oxides (Figure 1), it is possible to observe that the material exhibits the presence of metallic titanium, coming from the metallic base, and also the presence of films in the tetragonal phase of RuO_2_ and in the rutile phase TiO_2_. According to the analysis, it is not possible to observe the presence of SnO_2_ and Ta_2_O_5_. However, according to the Hume-Rothery rules, in substitutional solid solutions, the atomic size factor establishes that the difference between the atomic rays of two elements cannot exceed 15% [30], which occurs when we are considering the ions used for the electrode (Ru^4+^, Ti^4+^, Sn^4+^, and Ta^5+^) according to Table 1. Thus, it is possible to say that Sn^4+^, and Ta^5+^ ions were also present in the electrode.

Using XRD data, it was also possible to calculate the average crystallite size for the different crystalline planes of RuO_2_ and TiO_2_, using the Scherrer equation [31]:

D = 0.9 × λ/β × cos θ_β_ (2)

where D is the average size of the crystallite, λ the wavelength of the radiation, β the width of the reflection in radians and θ_β_ the angle of maximum intensity of the reflection. The results are shown in Table 2.

From the data obtained in Table 2, it is noted that lower values were obtained for Ti/RuO_2_-TiO_2_-SnO_2_-Ta_2_O_5_ (30:50:10:10), which according to Cheng et al. could provide better electronic and photocatalytic properties [32]. Knowing that better electronic and photocatalytic properties are possible when smaller crystallite radii are found, it is expected that the quaternary electrodes of composition Ti/RuO_2_-TiO_2_-SnO_2_-Ta_2_O_5_ in both studied proportions will obtain promising results when compared to the standard Ti/TiO_2_RuO_2_ (70:30).

#### 3.1.2. Scanning Electron Microscopy

**Figure 2:**
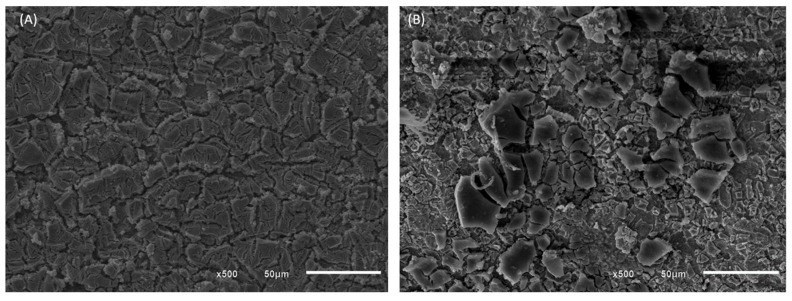
SEM images of (**A**) DSA of Ti/RuO_2_-TiO_2_-SnO_2_Ta_2_O_5_ (30:40:10:20) composition and (**B**) Ti/RuO_2_TiO_2_ (30:70) composition, in 500× magnification.

The analysis of the physical characteristics of the DSA electrodes of composition Ti/TiO_2_RuO_2_ and Ti/RuO_2_-TiO_2_-SnO_2_-Ta_2_O_5_ was performed by scanning electron microscope, SEM. This equipment is widely used for microstructural analysis of solid materials and images can be obtained with magnifications of up to 100,000 times and resolutions of up to 20 nm [32,33]. In Figure 2, the morphology of the materials is presented at 500× magnification, obtained through SEM, it is evident that the surfaces of both materials have structure considered by the literature similar to “mud cracked”, typical for DSAs prepared by the thermal decomposition method. In this morphology, the cracks designated the macro roughness, while the pores exhibited the micro-roughness [19].

### 3.2. Electrochemical Characterization

#### 3.2.1. Cyclic Voltammetry

**Figure 3:**
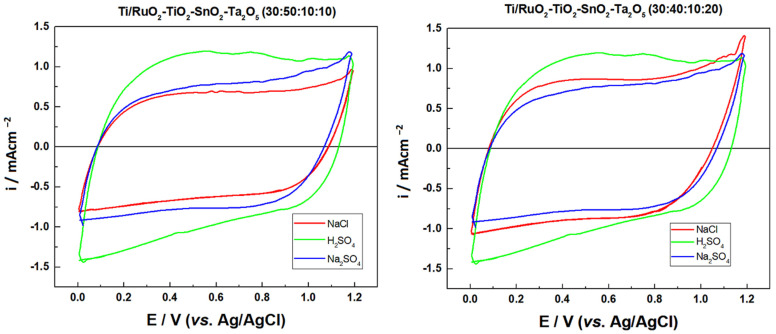
Cyclic voltammograms obtained for the electrode composition Ti/RuO_2_-TiO_2_-SnO_2_Ta_2_O_5_ (30:40:10:20) and Ti/RuO_2_-TiO_2_-SnO_2_-Ta_2_O_5_ (30:50:10:10), with the electrolytes: Na_2_SO_4_ 0.05 mol L^−1^ (−), H_2_SO_4_ 0.05 mol L^−1^ (−), and NaCl 0.167 mol L^−1^ (−). Conditions: v = 0.05 V s^−1^, potential range: 0.00 to 1.20 V vs. Ag/AgCl.

The electrochemical behavior of the previously prepared DSAs was investigated via cyclic voltammetry, the following compositions were tested: Ti/RuO_2_-TiO_2_-SnO_2_Ta_2_O_5_ (30:40:10:20) and Ti/RuO_2_-TiO_2_-SnO_2_Ta_2_O_5_ (30:50:10:10) in duplicate, so the first tests were carried out to find the electrolyte with the highest charge. The electrolytes tested were: Na_2_SO_4_ (0.05 mol L^−1^), NaCl (1.67 mol L^−1^), and H_2_SO_4_ (0.5 mol L^−1^), so that the concentrations were defined so that all solutions had the same ionic strength (μ = 0.25). In this way, the values between the anodic (qa) and cathodic (qc) charge densities were calculated based on Figure 3, as shown in Table 3.

Figure 3 illustrates the behavior of the Ti/RuO_2_-TiO_2_-SnO_2_Ta_2_O_5_ (30:40:10:20) and Ti/RuO_2_-TiO_2_-SnO_2_Ta_2_O_5_ (30:50:10:10) electrodes. In the investigated potential range (0.00 to 1.20 V vs. Ag/AgCl), it is possible to observe in all voltammograms an increase in the current response by 1.1 V vs. Ag/AgCl which is associated with the oxygen evolution reaction (OER) of water oxidation. In the region of 0.10 to 0.70 V vs. Ag/AgCl, it is possible to observe a peak attributed to the Ru(III)/Ru(IV) redox transition, this is more visible in the composition sample (30:50:10:10) with H_2_SO_4_ electrolyte. It is also possible to observe the second peak in the region between 0.8–1.1 V vs. Ag/AgCl, assigned to Ru(IV)/Ru(VI) redox transition [18,34].

In the region of 1.0 V vs. Ag/AgCl, begins the chlorine evolution reaction and an increase in the current response is also observed, normally associated with the coexistence of the water oxidation [18]. However, the composition DSA (30:40:10:20) in Na_2_SO_4_ and NaCl medium overlap, which indicates that this peak must be attributed just to the oxidation of water. Finally, a slight increase in charge is observed in relation to the voltammogram when the Na_2_SO_4_ electrolyte is used, compared to NaCl and Na_2_SO_4_ under an identical ionic strength, which is also possible to observe in Table 1. Finally, it is also possible to observe that, except for sample two of Ti/RuO_2_-TiO_2_-SnO_2_Ta_2_O_5_ (30:40:10:20) when Na_2_SO_4_ 0.050 mol L^−1^ was used, all samples present their ratios between cathodic and anodic charge close to the unit value, which indicates a behavior close to reversible.

**Figure 4:**
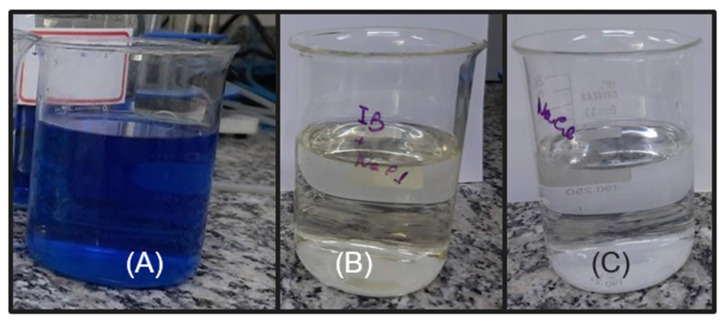
(**A**) IB solution (6 mg L^−1^) solubilized in H_2_SO_4_ + NaCl (0.167 mol L^−1^) before voltammetry; (**B**) solution with the same composition after cyclic voltammetry at 0.01 V s^−1^ and (**C**) solution with only NaCl (0.167 mol L^−1^).

The following experiments were carried out with the DSA of composition Ti/RuO_2_-TiO_2_-SnO_2_Ta_2_O_5_ (30:40:10:20), due to the high value of the cathodic and anodic charge when using NaCl as a supporting electrolyte. The electrolyte was chosen because when using NaCl, it was possible to notice that after some voltammetry the solution became translucent, as can be seen in Figure 4. The initial hypothesis is that the IB is oxidized by the presence of NaCl in the solution when a certain potential is applied. Since it is reported in the literature that when the NaCl salt is used as a supporting electrolyte, and an overpotential is applied in relation to the chloride reduction potential (Equation (3)), there is the generation of active chlorine species (Cl_2_, HClO, ClO^−^) within the solution so that these species are capable of promoting indirect oxidation of the dye in question [35,36]. The formation of active chlorine species occurs by the oxidation of the chloride anion (Cl^−^) at the anode, releasing chlorine (Cl_2_(aq)). As chlorine diffuses within the reaction, it is hydrolyzed, forming HClO and Cl^−^, as can be seen in Equations (4)–(6) [37].

Cl_2_(g) + 2e^−^ ⇌ 2Cl^−^(aq) E° at 25 °C = +1.36 V (3)

2Cl^−^(aq) → Cl_2_(aq) + 2e^−^ (4)

Cl_2_(aq) + H_2_O(l) → HClO(aq) + Cl^−^(aq) + H^+^(aq) (5)

HClO(aq) ⇌ H^+^(aq) + ClO^−^(aq) (6)

#### 3.2.2. Stability Test

**Figure 5:**
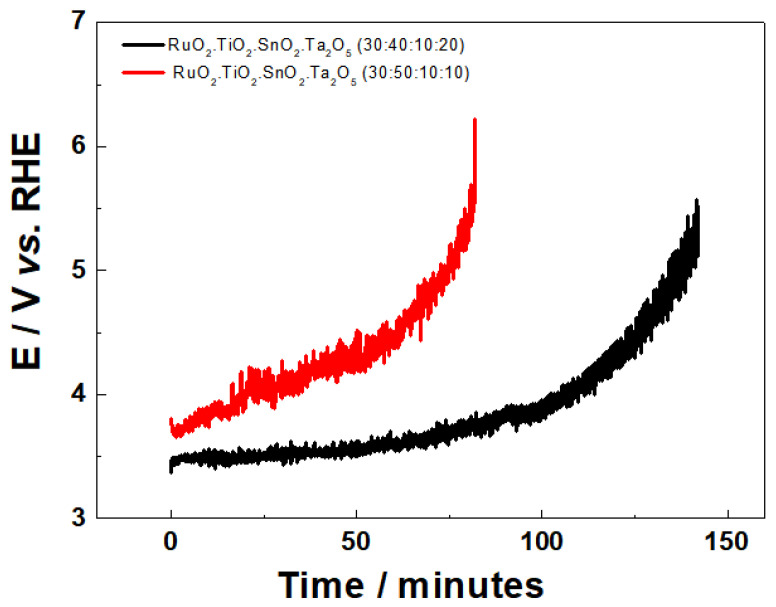
Potential curve behavior vs. time as a function of the electrode composition Ti/RuO_2_-TiO_2_-SnO_2_Ta_2_O_5_ (30:40:10:20) (−) and Ti/RuO_2_-TiO_2_-SnO_2_Ta_2_O_5_ (30:50:10:10) (−). Conditions: 0.5 mol L^−1^ H_2_SO_4_, j = 750 mA cm^−2^.

The stability of the Ti/RuO_2_-SnO_2_-TiO_2_-Ta_2_O_5_ system as a function of its composition was evaluated at a current density of 750 mA cm^−2^ in 0.5 mol L^−1^ H_2_SO_4_, considering the time required for the potential reaches 6 V vs. RHE, as shown in Figure 5 and Table 4. Once above this potential, DSA is considered inactive, due to the total degradation of the active layer of the deposited oxide, or by the formation of a TiO_2_ film in the metallic Ti layer/active layer [38].

In Figure 5, the DSA of composition Ti/RuO_2_-TiO_2_-SnO_2_Ta_2_O_5_ (30:50:10:10), presented a potential variation as a function of time slower than the DSA (30:40:10:20), indicating it to be more susceptible to extreme conditions. Regarding the binary composition Ti/RuO_2_-TiO_2_ (30:70), it is possible to find in the literature good durability of the material [39,40].

### 3.3. Indigo Blue Dye Degradation Assays

#### 3.3.1. Electrocatalysis

**Figure 6:**
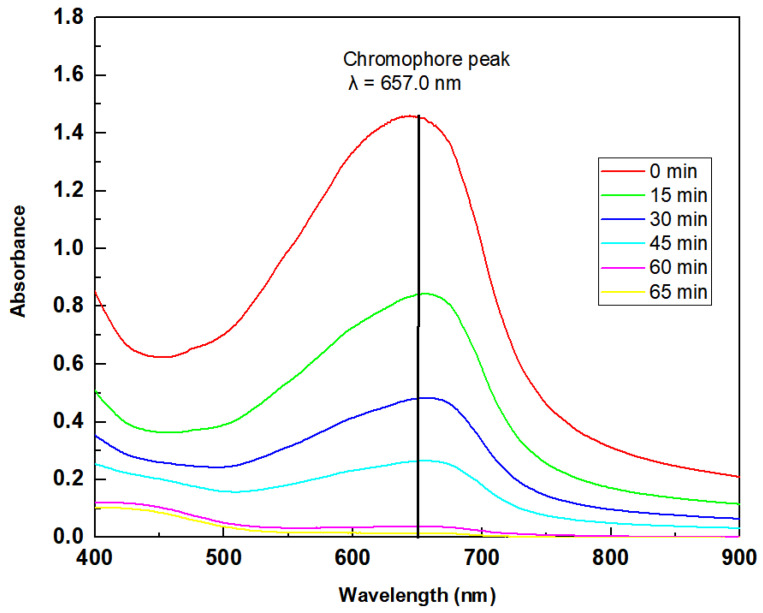
UV-Vis spectrum of the solution containing IB. Effect of photoelectrocatalysis on the absorption spectra in the UV-Vis region of the IB dye and UV irradiation. Condition: Δλ = 400–900 nm, i = 20 mA, UV light λ = 365 nm, C_IB_ = 100 mg L^−1^, C_NaCl_ = 0.167 mol L^−1^, T = 25 °C.

The electrocatalysis assay was performed with a DSA of composition Ti/RuO_2_-TiO_2_-SnO_2_Ta_2_O_5_ (30:40:10:20) which was compared to the standard Ti/RuO_2_-TiO_2_ (30:70). The degraded solution consisted of IB at a concentration of 100 mg L^−1^, with 2 mL of concentrated sulfuric acid, used to solubilize the dye and NaCl electrolyte 0.167 mol L^−1^. In Figure 6, it is possible to observe the UV-Vis spectrum of the solution in the visible wavelength region.

**Figure 7:**
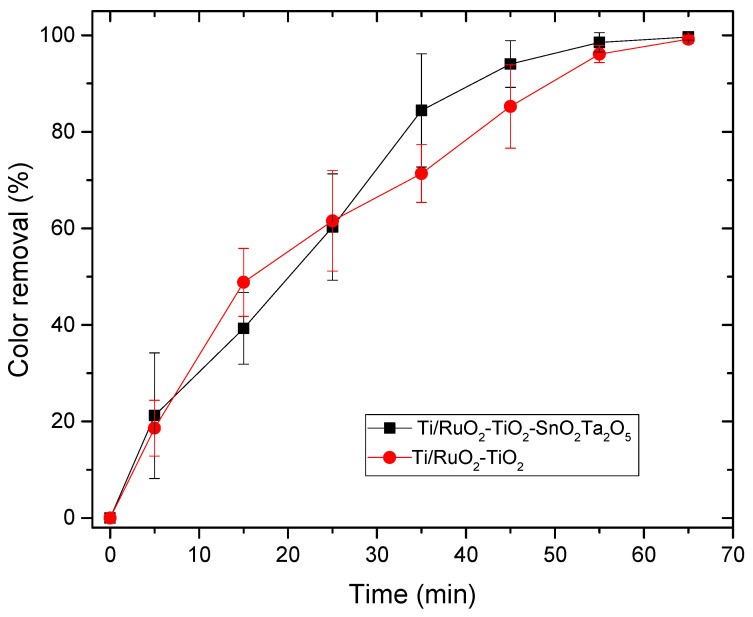
Relationship between color removal and time in the electrocatalysis process for the DSAs Ti/RuO_2_-TiO_2_-SnO_2_-Ta_2_O_5_ (30:40:10:20) and Ti/RuO_2_-TiO_2_ (30:70). Condition: i = 20 mA, C_IB_ = 100 mg L^−1^, C_NaCl_ = 0.167 mol L^−1^, T = 25 °C.

The maximum lambda found for the IB was 657.0 nm, with this data the calibration curve was performed in the UV-Vis, and this curve was used to determine the concentration of the solution as a function of the reaction time. The test was performed with the two DSAs in triplicate, with a current of 20 mA supplied by the AFR source. The results obtained are shown in Figure 7.

During the electrocatalysis process, it was possible to observe the formation of bubbles on the surface of the electrode, due to the reaction of the detachment of oxygen and chlorine, which can negatively influence the reactive area of the DSA. However, based on the curve, the DSA obtained a good response, even with a low current (20 mA) and both tested anodes were able to remove 100% of the color of the IB dye in 65 min, demonstrating that the anodic oxidation was effective for the removal of color background. As reported by Soni et al. (2020), the use of NaCl as an electrolyte provides indirect oxidation, in addition, as sulfuric acid is being used in the dilution of IB, it is an acidic medium, which is also favorable for a high color reduction with electrodes like DSA by indirect oxidation [41]. When comparing the performance of the electrodes, it is possible to observe that at the beginning of the reaction the standard Ti/RuO_2_-TiO_2_ (30:70) obtained a better performance, until it matched the performance of the composition Ti/RuO_2_-TiO_2_-SnO_2_Ta_2_O_5_ (30:40:10:20) in 25 min, and from that moment the quaternary electrode started to perform better when compared to the standard. The good performance with a standard electrode was already expected because it is a widely used composition, with 70% TiO_2_ which is a catalyst with great light absorption properties [15] and 30% of RuO_2_, a promising catalyst for hydrogen evolution reaction [42]. On the other hand, the composition of the quaternary anode also presents Ta_2_O_5_ with excellent properties as a semiconductor and stabilizer [20], but its application has been little studied in DSAs and SnO_2_ with a significant electrooxidation capacity and corrosion stability [23]. In this way, when used together, it was possible to obtain a competitive result with the standard formulation.

#### 3.3.2. Photocatalysis

**Figure 8:**
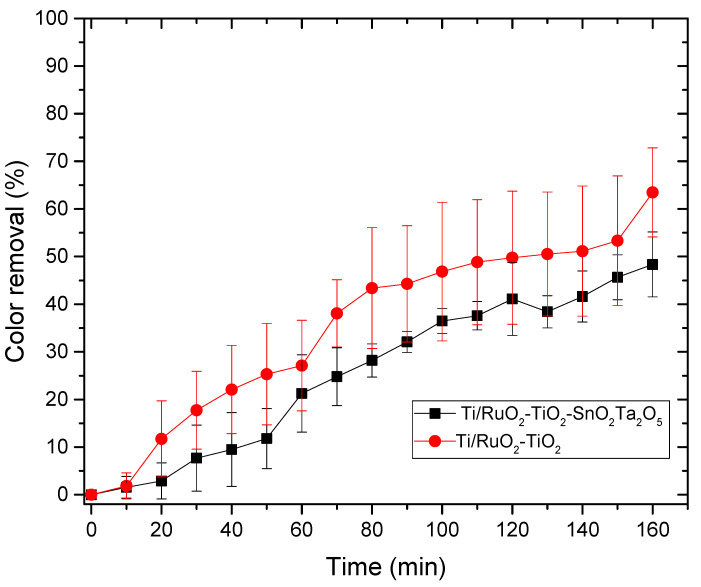
Relationship between color removal and time in the photocatalysis process for the DSAs Ti/RuO_2_-TiO_2_-SnO_2_Ta_2_O_5_ (30:40:10:20) and Ti/RuO_2_-TiO_2_ (30:70). Condition: UV light λ = 365 nm, C_IB_ = 100 mg L^−1^, C_NaCl_ = 0.167 mol L^−1^, T = 25 °C.

For the photocatalysis assay with the same DSAs of composition Ti/RuO_2_-TiO_2_-SnO_2_Ta_2_O_5_ (30:40:10:20) and Ti/RuO_2_-TiO_2_ (30:70), in addition to the same solution of AI 100 mg L^−1^, with 2 mL of concentrated sulfuric acid, used to solubilize the dye and NaCl electrolyte 0.0167 mol L^−1^. The test was performed in triplicate and the results obtained are shown in Figure 8.

As the color removal process by photocatalysis was inefficient, the time was limited to 3 h. It is possible to observe that in the photocatalysis DSA Ti/RuO_2_-TiO_2_ (30:70), type as standard, more effective color removal was obtained when compared to the new DSA formulation tested (Ti/RuO_2_-TiO_2_-SnO_2_Ta_2_O_5_), showing that standard DSA has a better response to UV light when compared to quaternary composition. This is due to the higher proportion of TiO_2_ in the second electrode, which is a material that has great light absorption properties and, due to this characteristic, it is currently widely applied to photocatalytic and antibacterial technology [15].

#### 3.3.3. Photoelectrocatalysis

**Figure 9:**
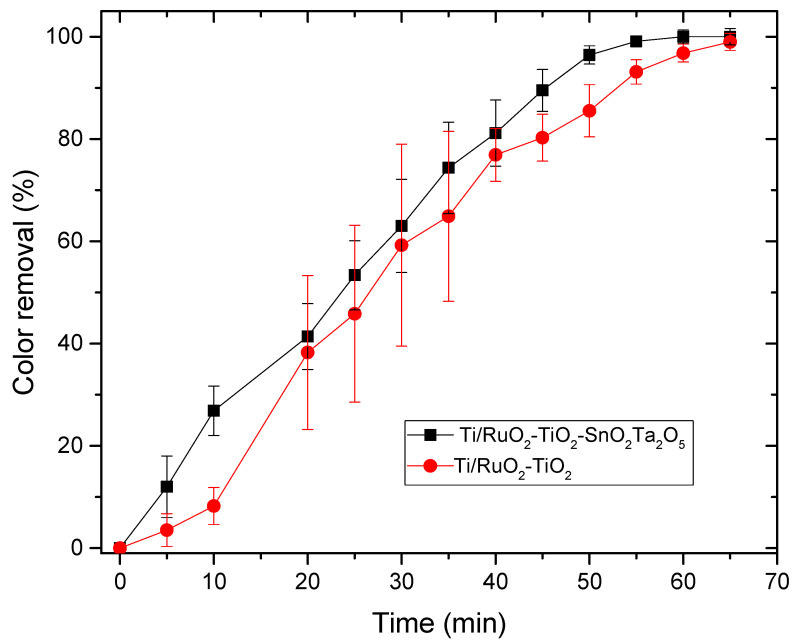
Relationship between color removal and time elapsed in the photoelectrocatalysis process for the DSAs Ti/RuO_2_-TiO_2_-SnO_2_Ta_2_O_5_ (30:40:10:20) e Ti/RuO_2_-TiO_2_ (30:70). Condition: UV light λ = 365 nm, i = 20 mA, C_IB_ = 100 mg L^−1^, C_énacle_ = 0.167 mol L^−1^, T = 25 °C.

The photoelectrocatalysis assay was performed with the same DSAs of composition Ti/RuO_2_-TiO_2_-SnO_2_Ta_2_O_5_ (30:40:10:20) and Ti/RuO_2_-TiO_2_ (30:70), and the same solution of IB 100 mg L^−1^, with 2 mL of concentrated sulfuric acid, and a supporting electrolyte NaCl 0.0167 mol L^−1^. The test was performed in triplicate and the results obtained are shown in Figure 9.

**Figure 10:**
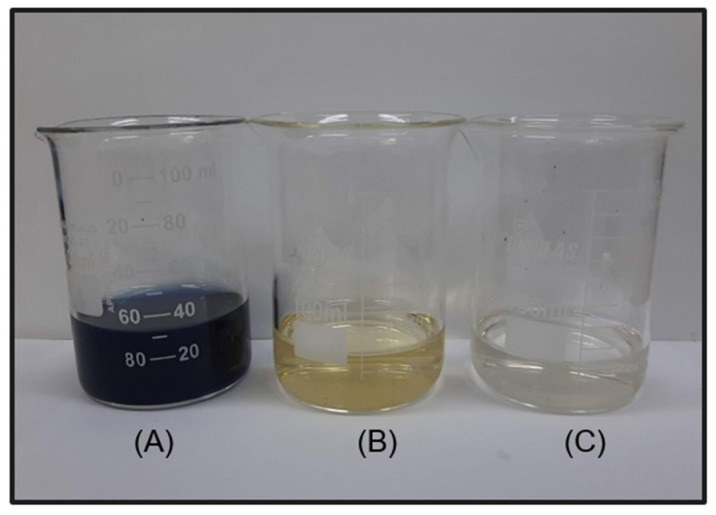
(**A**) solution of IB (100 mg L^−1^) solubilized in H_2_SO_4_ in NaCl (0.167 mol L^−1^); (**B**) the same solution after the photoelectrocatalysis process for 65 min, and (**C**) solution after photoelectrocatalysis and addition of Na_2_SO_3_.

For the photoelectrocatalysis test, the performance of DSA Ti/RuO_2_-TiO_2_-SnO_2_Ta_2_O_5_ (30:40:10:20) was better than the standard, since a color removal of 100% in 60 min was achieved; however, only 5 min later the DSA Ti/RuO_2_-TiO_2_ (30:70) achieved 100% color removal. When combining UV irradiation with the current, the quaternary formulation again obtained competitive results, since, as mentioned, SnO_2_ has significant electrooxidation and Ta_2_O_5_ acted as a stabilizer. In Figure 10, it is possible to observe the color of the solution before and after 65 min of photoelectrocatalysis, the final solution has a yellowish color.

As the initial suspicion is that the characteristic color is due to the presence of chlorinated compounds from the electrolyte (Equations (3)–(6)), sodium sulfide was used in this final solution. When reacting with chlorine and water, HCl was formed (Equation (7)), with translucent color, as can be seen in Figure 10, which confirms that the yellow color is due to the presence of chlorine in the solution.

Cl_2_(aq) + Na_2_SO_3_(aq) + H_2_O(l) → Na_2_SO_4_(aq) + 2HCl(aq) (7)

Figure 6 shows the UV-vis spectrum of the initial solution of IB dye 100 mg L^−1^ and after different reaction times. The IB dye has only one peak in the visible region, at λ = 657 nm, and Figure 6 shows that in the first 15 min of reaction there is a decrease in this characteristic band, which continues to become smaller and smaller until it is no longer possible to be observed after 60 min. At that time, the absorbance of the solution was 0.007 and the color removal was 100%. At the time of 60 and 65 min, it is also possible to observe the formation of a new peak, close to 430 nm, characteristic of the yellow color that the solution takes on at that time due to the formation of chlorinated compounds. Other techniques have already been tested in the removal of IB, Table 5 presents some works and the results obtained in this article. Among the techniques used, the use of fungi stands out, which, in addition to being environmentally friendly, presented 100% color removal; on the other hand, for the removal to be complete, it took 4 days. Another technique that stands out is adsorption, because it uses a high initial concentration and obtains a removal of 90%; however, adsorption consists only of the transfer of mass from the fluid phase to the surface of the solid, allowing the separation of the components of the fluid, but not its degradation. In this work, the techniques of photocatalysis, electrocatalysis, and photoelectrocatalysis were explored using the DSA Ti/RuO_2_-TiO_2_-SnO_2_Ta_2_O_5_ (30:40:10:20) compared to Ti/RuO_2_-TiO_2_ (30:70). Photocatalysis did not perform well, as it took 3 h to remove just 63% of the dye, whereas the electrocatalysis and photoelectrocatalysis techniques had an excellent performance when degrading 100% of the IB at a concentration of 100 mg L^−1^ at 65 and 60 min, respectively, using a current of 20 mA. It is important to highlight that in the last two techniques mentioned, the performance of the new formulation was very similar to the standard composition of the electrode, demonstrating that the new composition is competitive, as well as interesting because they bring into their formulation SnO_2_ and Ta_2_O_5_, which, as mentioned, are materials with good stability. However, the new composition is still in the testing phase and therefore more in-depth studies are needed.

## 4. Conclusions

With the data obtained, it is possible to say that the DSA of composition Ti/RuO_2_-TiO_2_-SnO_2_Ta_2_O_5_ (30:40:10:20) has promising results in the degradation of the IB dye, especially when applying a current, even if low. Since the results found when the photoelectrocatalysis technique was applied, with a current of 20 mA and UV irradiation, were the best, with a color removal of 100% in 60 min. However, electrocatalysis obtained similar results, when applying at the current of 20 mA with a removal also of 100% in 65 min. Color removal when using photocatalysis with both DSAs was unsatisfactory, with maximum removal of 48% when using the quaternary anode. When comparing the results of the standard DSA and Ti/RuO_2_-TiO_2_-SnO_2_Ta_2_O_5_, it is possible to affirm that the new composition performed well, with results that just did not stand out when using photocatalysis. Thus, the data obtained show the applicability of DSA Ti/RuO_2_-TiO_2_-SnO_2_Ta_2_O_5_ (30:40:10:20), which is shown to be an effective activity for removing color from textile effluents such as IB from the aqueous medium.

## Figures and Tables

**Table 1:** The ionic radius of ions Ru^4+^, Ti^4+^, Sn^4+^, e Ta^5+^. Adapted from Shannon (1976).

Ion	Ionic Radius (nm)
Ru^4+^	0.062
Ti^4+^	0.056
Ta^5+^	0.068
Sn^4+^	0.069

**Table 2:** Average crystallite size obtained for the tetragonal RuO_2_ and TiO_2_ phases in different diffraction planes.

Electrode Composition			Average Crystallite Size (nm)						
Nominal	Experimental (EDX)	101	110	111	202	211	310	321	400
Ti/RuO_2_-TiO_2_-SnO_2_-Ta_2_O_5_ (30:50:10:10)	Ti/RuO_2_-TiO_2_-SnO_2_-Ta_2_O_5_ (40:32:6:21)	-	-	26	77	39	-	54	-
Ti/RuO_2_-TiO_2_-SnO_2_-Ta_2_O_5_ (30:40:10:20)	Ti/RuO_2_-TiO_2_-SnO_2_-Ta_2_O_5_ (46:32:5:17)	-	27	40	77	39	-	82	-
Ti/RuO_2_-TiO_2_ (30:70)	Ti/RuO_2_-TiO_2_ (21:79)	35	28	-	-	54	-	-	86

**Table 3:** Calculated values of the relationships between anodic and cathodic charge densities for the DSAs. Conditions: v = 0.01 V s^−1^, potential range: 0.00 to 1.20 V vs. Ag/AgCl.

Electrode	qa/C m^−2^	qc/C cm^−2^	qa/qc
**H_2_SO_4_ 0.050 mol L^−1^**			
Sample 1—Ti/RuO_2_-TiO_2_-SnO_2_-Ta_2_O_5_ (30:40:10:20)	19.0	22.6	0.843
Sample 2—Ti/RuO_2_-TiO_2_-SnO_2_-Ta_2_O_5_ (30:40:10:20)	10.0	11.3	0.881
Sample 1—Ti/RuO_2_-TiO_2_-SnO_2_-Ta_2_O_5_ (30:50:10:10)	16.5	18.9	0.873
Sample 2—Ti/RuO_2_-TiO_2_-SnO_2_-Ta_2_O_5_ (30:50:10:10)	21.8	25.1	0.868
**NaCl 0.167 mol L^−1^**			
Sample 1—Ti/RuO_2_-TiO_2_-SnO_2-_Ta_2_O_5_ (30:40:10:20)	15.4	21.6	0.712
Sample 2—Ti/RuO_2_-TiO_2_-SnO_2_Ta_2_O_5_ (30:40:10:20)	16.9	19.0	0.890
Sample 1—Ti/RuO_2_-TiO_2_-SnO_2_-Ta_2_O_5_ (30:50:10:10)	11.7	14.2	0.823
Sample 2—Ti/RuO_2_-TiO_2_-SnO_2_-Ta_2_O_5_ (30:50:10:10)	13.1	16.0	0.817
**Na_2_SO_4_ 0.050 mol L^−1^**			
Sample 1—Ti/RuO_2_-TiO_2_-SnO_2_Ta_2_O_5_ (30:40:10:20)	12.2	14.5	0.835
Sample 2—Ti/RuO_2_-TiO_2_-SnO_2_Ta_2_O_5_ (30:40:10:20)	9.42	13.9	0.674
Sample 1—Ti/RuO_2_-TiO_2_-SnO_2_Ta_2_O_5_ (30:50:10:10)	10.8	10.8	0.994
Sample 2—Ti/RuO_2_-TiO_2_-SnO_2_Ta_2_O_5_ (30:50:10:10)	10.2	15.5	0.656

**Table 4:** Comparison of ADEs in relation to stability. Conditions: 0.5 mol L^−1^ H_2_SO_4_, j = 750 mA cm^−2^.

Electrode	Time (min)
Ti/RuO_2_-TiO_2_-SnO_2_Ta_2_O_5_ (30:40:10:20)	82
Ti/RuO_2_-TiO_2_-SnO_2_Ta_2_O_5_ (30:50:10:10)	142

**Table 5:** Comparison between techniques used to remove IB dye from aqueous medium.

Technique	C_0_ (IB)	Conditions	Color Removal	Reference
Adsorption	100 mg L^−1^	30 min, 3 g de vermicompost, 1000 rpm, 25 °C	90%	6
Ligninolytic basidiomycete fungi	-	Fungi *Phellinus gilvus* 4 days, 25–30 °C	100%	26
Photocatalysis + Fenton reagent	25 mg L^−1^	45 min, UV light, Fenton reagents	-	27
Electrochemical discoloration	26 mg L^−1^	60 min, 5 V, pH 4.5, 25 °C	90%	28
Photocatalysis	100 mg L^−1^	3 h, UV light, 25 °C	63%	Present work
Electrocatalysis	100 mg L^−1^	65 min, 20 mA, 25 °C	100%	Present work
Photoelectrocatalysis	100 mg L^−1^	60 min, luz UV, 20 mA, 25 °C	100%	Present work

## Data Availability

Not applicable.
